# The effectiveness of a mobile therapeutic application in coping with stress and burnout

**DOI:** 10.1192/j.eurpsy.2023.395

**Published:** 2023-07-19

**Authors:** E. Wojtyna, A. Mucha

**Affiliations:** Institute of Psychology, University of Silesia, Katowice, Poland

## Abstract

**Introduction:**

Excessive stress at work is a problem that leads to numerous complications, including the development of depression and burnout. A very important factor contributing to coping is a change in attitude to the situation at work. A helpful tool is Cognitive Behavioral Therapy. However, access to CBT is limited due to cost and lack of time. Mobile therapeutic application based on CBT may be the answer to these barriers.

**Objectives:**

The aim of the study was to test the effectiveness of mobile CBT in comparison with CBT in the face to face formula and in comparison with the control group, not receiving any intervention.

**Methods:**

The face-to-face (ftfCBT) CBT intervention included 12 hour treatment sessions. Mobile CBT (mCBT; *UpBalance* smartphone application) included a therapeutic program analogous to the protocol used in the ftfCBT group. The content of the application was divided into short educational parts (in the form of videos, animations, articles and podcasts) and exercise parts available to the subject throughout the duration of the study. The study involved 90 subjects randomly assigned to three groups: ftfCBT, mCBT and control (randomization 1: 1: 1). Two measurements were made - baseline and after 12 weeks. The following questionnaire methods were used: the Thermometer of Distress, the Occupational Stress Questionnaire and the LBQ to measure burnout.

**Results:**

In the initial measurement, no differences were observed between the ftfCBT, mCBT and control groups. After 12 weeks in the control group, there were no differences between the t0 and t1 measurements. In the ftfCBT and mCBT groups, an improvement was observed in both the reduction of the level of distress and the reduction of burnout symptoms. There were no differences in t1 between the ftfCBT and mCBT groups. A higher level of compliance was observed in the mCBT group than in the ftfCBT group.

**Conclusions:**

A mobile therapeutic application focused on coping with occupational stress is an effective intervention improving the mental state of employees. Mobila digital cognitive behavioral therapy can also be a helpful alternative to classic psychotherapy and can respond to the unmet needs of employees in terms of access to therapy at a suitable time.

**Disclosure of Interest:**

E. Wojtyna Grant / Research support from: National Centre for Reaserch and Development, A. Mucha: None Declared

